# A Wearable Leg Support for Surgical Ergonomics in Knee Arthroscopy

**DOI:** 10.1016/j.eats.2025.103879

**Published:** 2025-09-25

**Authors:** Jacob S. Luttjeboer, Duncan E. Meuffels, Wybren A. van der Wal

**Affiliations:** a Department of Orthopaedic Surgery, Bergman Clinics, Breda, The Netherlands; b Department of Orthopaedics and Sports Medicine, Erasmus MC, University Medical Center Rotterdam, Rotterdam, The Netherlands; c Sports Valley, Department of Orthopaedic Surgery, Gelderse Vallei Hospital, Ede, The Netherlands

## Abstract

The Knee Arthroscopy Belt (Knee Arthroscopy Belt B.V.) is an ergonomic device designed to reduce lower back strain and optimize lower limb positioning during knee arthroscopy, eliminating the need for a surgical footrest and reducing the need for manual leg support by operating room personnel. Surgeons often experience low back pain due to prolonged, uncomfortable static postures. The Knee Arthroscopy Belt, worn under the sterile gown, consists of a leather belt and neoprene-covered aluminum brackets that support the patient’s lower leg. It promotes a horizontal pelvis position for the surgeon, allowing the application of varus and valgus stresses with much less effort, compared to the standard technique, where the patient’s lower leg is on the upper leg or the iliac crest of the surgeon. The Knee Arthroscopy Belt also enhances lumbar spine stabilization, mimicking the benefits of a powerlifting belt. The Knee Arthroscopy Belt represents a promising development in surgical ergonomics, potentially improving efficiency and reducing musculoskeletal strain when performing knee arthroscopy.

Orthopaedic surgeons practice a physically demanding profession, which can frequently involve work-related complaints.^1^, ^2^, ^3^, ^4^, ^5^, ^6^, ^7^ Among other things, low back pain is frequent. Swank et al.^1^ describe a worldwide prevalence in the general population of 2% to 25%. Among orthopaedic surgeons, occurrence rates of 35% to 66% are reported.^1^^,^^3^, ^4^, ^5^, ^6^

During and after performing knee arthroscopy programs using a surgical footrest to support 1 foot, the authors frequently experienced lower back pain, resulting from the prolonged static and uncomfortable positions during surgery. Wanting to find out if there is a better way to unload the surgeon’s lumbar spine and have more control over the patient’s lower leg, the authors (J.S.L., W.A.v.d.W)^,^ have developed and patented the Knee Arthroscopy Belt (Knee Arthroscopy Belt B.V.) with leg supports. The purpose of this tool is to improve ergonomics while performing knee arthroscopy. The Knee Arthroscopy Belt is a lightweight, low-profile device that is worn under the surgical gown around the waist. It allows us to operate with a horizontal pelvis instead of a tilted pelvis while giving varus/valgus stress to the knee. We no longer need a surgical footrest, and the leg is much more stable on the aluminum leg support of the belt. Moreover, giving varus/valgus stress to the knee is kept more easily and requires less effort, thus minimizing fatigue. The Knee Arthroscopy Belt can also be used for knee arthroscopy with the patient’s leg on the table. Low back complaints that we previously experienced decreased significantly. The authors are currently preparing a prospective clinical study with a crossover design, in which a group of orthopaedic surgeons will evaluate the Knee Arthroscopy Belt during routine knee arthroscopies. This Technical Note describes the Knee Arthroscopy Belt and its application in arthroscopic knee surgery.

## Surgical Technique

**Fig 1:**
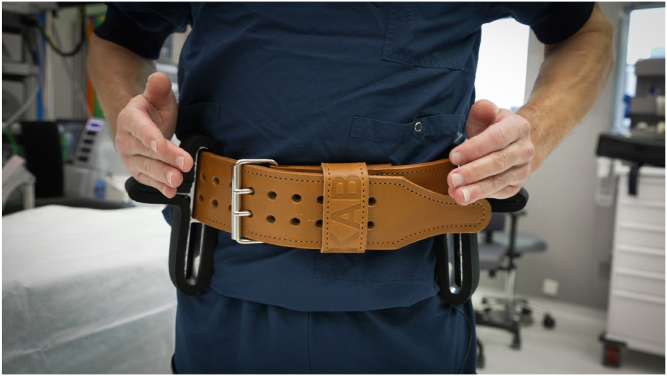
Surgeon wearing the Knee Arthroscopy Belt. The hands of the surgeon show where the patient’s lower leg is positioned on the leg supports. The buckle of the belt should be in the middle between the leg supports. The free tapered end of the strap should not protrude beyond the leg support.

The Knee Arthroscopy Belt (Fig 1) is a patented Medical Device Regulation class 1 noninvasive medical device. It consists of an adjustable 7.6-cm-wide and about 10-mm-thick leather belt and 2 neoprene-covered aluminum brackets, which function as leg supports for the patient’s lower leg. The brackets are completely covered with a 10-mm neoprene layer, which distributes pressure and prevents tissue damage. The overhang of the neoprene layer on both sides of the brackets is 10 mm. Additionally, the neoprene layer helps prevent perforation of the sterile gown.

This belt is comparable to a powerlifting belt, worn around the waist just below the umbilicus. During strength efforts, the belt helps stabilize the lumbar spine and allows for better posture of the surgeon. The principle of a powerlifting belt is that it can help increase intra-abdominal pressure, stabilize the spine, reduce spinal compression, and reduce the risk of spinal injury during strength training.^8^^,^^9^

**Fig 2:**
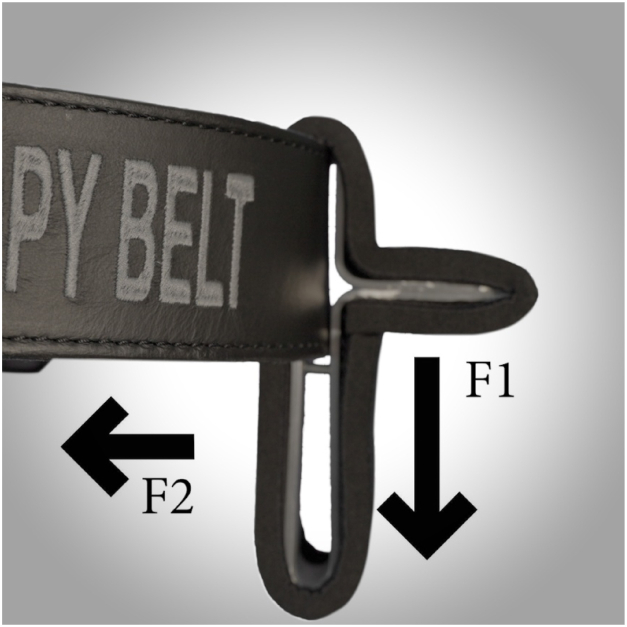
Image showing the principle of the Knee Arthroscopy Belt with its leg supports. Downward pressures from the patient’s foot/ankle on the leg support are minimized due to the leverage function of the lower arm of the leg support. The surgeon’s hip/pelvis helps stabilize the leg support, together with the strength of the belt itself.

The Knee Arthroscopy Belt is designed to relieve the lumbar spine of the surgeon and stabilize the patient’s lower leg better during surgery. This allows the surgeon to apply varus and valgus stress to the patient’s knee in a more controlled way. In addition, the downward forces of the patient’s leg are partially neutralized (Fig 2).

The belt should be worn tightly enough to resist forces during varus or valgus stress. The belt loses its stabilizing effect on the lumbar spine if it is worn too loosely.^8^^,^^9^

The upper edge of the belt should be positioned at, or slightly below, the level of the surgeon’s umbilicus (Fig 1).

Ideally, the belt buckle is positioned centrally between the 2 neoprene-covered aluminum brackets, with the end of the strap not extending past the brackets (Fig 1).

The supports can be slid along the strap as desired. The ideal positions are approximately at 10:00 and 14:00 (the surgeon’s umbilicus being 12:00) (Fig 1). In smaller patients, it is advisable to slide the supports a bit more anteriorly.

**Fig 3:**
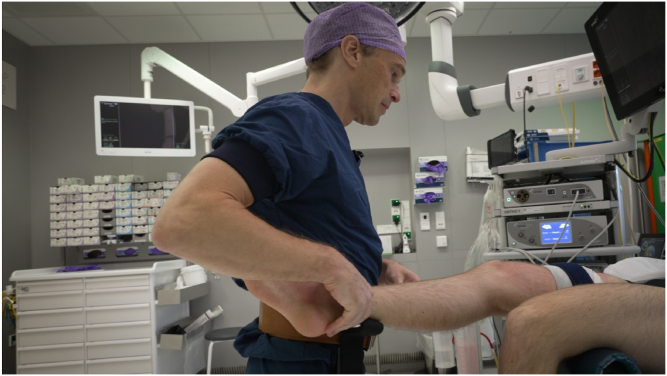
Surgeon wearing the Knee Arthroscopy Belt. The lower leg of the patient is put on the right-sided leg support to perform varus stress in order to open the lateral compartment.

**Fig 4:**
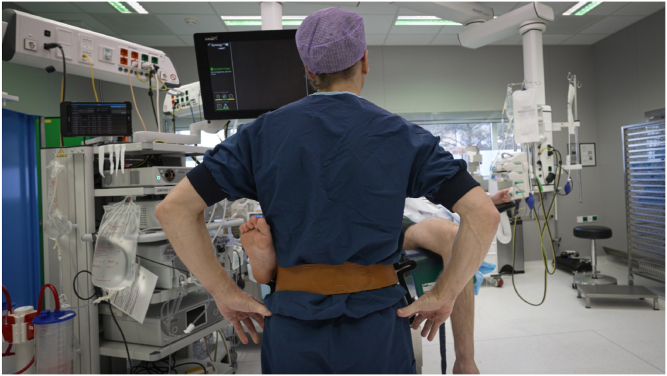
Surgeon wearing the Knee Arthroscopy Belt. The right foot/ankle is on the left-sided leg support. The surgeon can stand relaxed in this position. In order to open the medial compartment, valgus stress is given.

**Fig 5:**
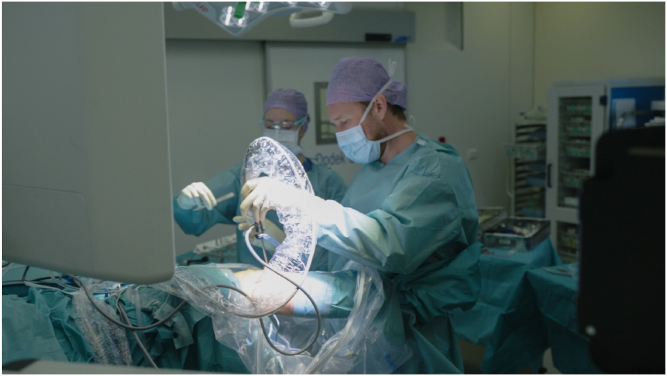
Surgeon performing arthroscopy of a right knee. The foot/ankle of the patient is positioned on the left leg support of the Knee Arthroscopy Belt in order to work in the medial compartment.

**Fig 6:**
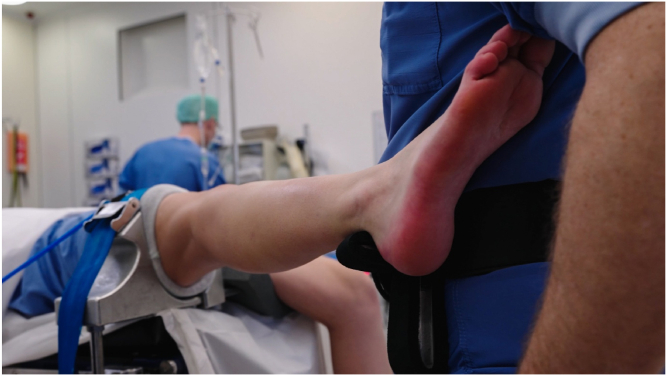
Surgeon wearing the Knee Arthroscopy Belt. The patient’s lower leg is put on the left-sided leg support.

The position of the patient’s foot/ankle on the brackets should be approximately at the level of the upper ankle joint. During varus stress, this is the Achilles tendon/lateral malleolus/ventrolateral side of the ankle at the level of the upper ankle joint. During valgus stress, this is the Achilles tendon/medial malleolus (Fig 3, Fig 4, Fig 5, Fig 6).

**Fig 7:**
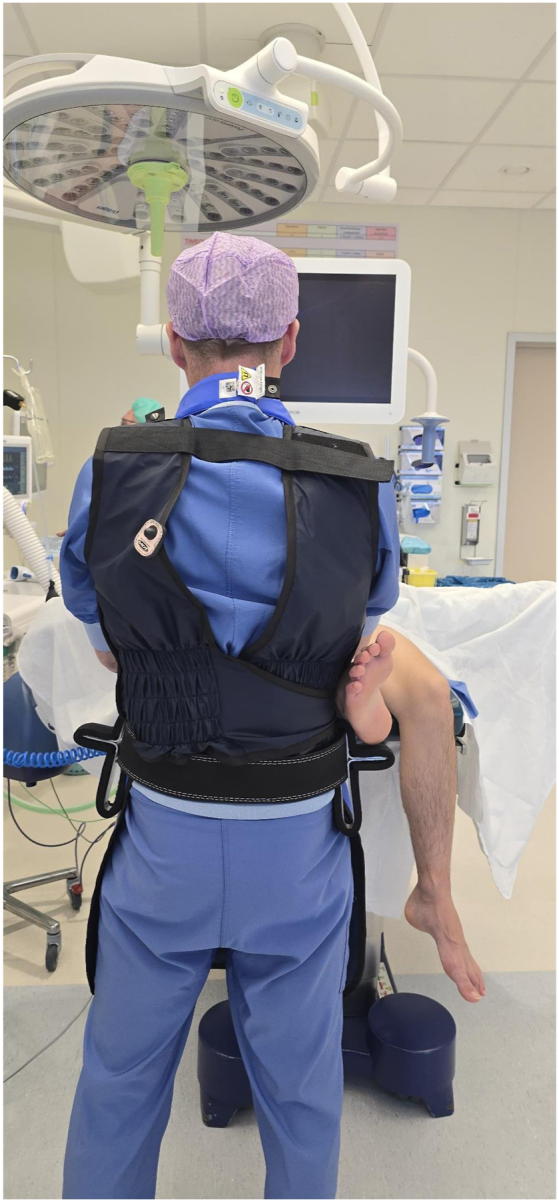
Surgeon wearing the Knee Arthroscopy Belt over a lead apron. Leg support while wearing a lead apron can be challenging. The Knee Arthroscopy Belt makes performing knee arthroscopy while wearing a lead apron easier.

To optimally benefit from the ergonomic added value of the knee arthroscopy belt, it is important to perform surgery with the pelvis in a horizontal position. Varus and valgus stress can be applied on the patient’s knee by moving in a horizontal plane while moving the table and/or a mechanical leg support vertically to further open or close the medial/lateral compartment of the knee.

Stabilizing the patient’s foot/ankle when put on the leg support of the Knee Arthroscopy Belt is performed by a slight push to the lower part of the bracket with your hip/pelvis. During surgery, stabilization of the bracket can be improved by standing in a snowboarder’s position with one foot somewhat in front of the other and your hip turned toward the patient.

The degree of knee flexion can also be varied during surgery. When applying stress to the knee, it is important to determine which bracket position works best for you. Experience shows that they work best when positioned slightly more ventrolateral when the patient has short legs, and for longer legs, the supports can be placed further back. Varus stress is somewhat easier when the knee is slightly less flexed than usual. This is to maintain proper tension and position of the foot/ankle on the leg supports. Personal preferences will, of course, vary among surgeons.

Finally, it is possible to remove the Knee Arthroscopy Belt during surgery. So, when the support of the Knee Arthroscopy Belt is not desired anymore, but the procedure is not yet over (e.g., during an anterior cruciate ligament reconstruction), you can simply loosen and drop the Knee Arthroscopy Belt under your sterile gown. However, our experience shows that it is quite comfortable to wear the strap throughout the entire procedure. It is also possible to operate sitting down with this strap, for example, when obtaining a hamstring graft at the beginning of an anterior cruciate ligament reconstruction.

For additional reinforcement of the sterile jacket, an extra adhesive sterile cloth can be placed at the level of the Knee Arthroscopy Belt. However, during 2 years of testing, no perforations or tears of the sterile surgical gowns have occurred during procedures.

The Knee Arthroscopy Belt is suitable for all arthroscopic procedures of the knee, especially those that are performed in the medial or lateral tibiofemoral compartment. Getting and maintaining access to the posteromedial or posterolateral compartment of the knee is foremost easier to perform. Pearls and pitfalls for the safe and effective use of the Knee Arthroscopy Belt are summarized in Table 1.

**Table 1:** Pearls and Pitfalls

Pearls	Pitfalls
Position leg supports at 10:00 and 14:00 for optimal support (the surgeon’s umbilicus is 12:00).	Wearing the belt too loosely reduces lumbar support and the stabilizing function of the leg supports.
For optimal leverage function of the brackets, use lateral pressure from the hip/pelvis for additional stabilization (Fig 2).	Leg support pressure may cause discomfort on the iliac crest. It helps to slide the brackets a bit forward or backward to change pressure points.
Use table height to open the medial/lateral compartment. The leg supports will stabilize the patient’s lower leg when doing this.	Short learning curve may hinder early use. The Knee Arthroscopy Belt mandates a slight change in posture/technique compared to classic knee arthroscopy with a step stool.
Easy to use in combination with a lead apron (pediatric anterior cruciate ligament or other cases) (Fig 7). Make sure to have the right size Knee Arthroscopy Belt when doing this.	Removing the Knee Arthroscopy Belt during surgical procedures is possible but can be somewhat challenging.
Using the Knee Arthroscopy Belt for knee arthroscopy with the leg on the table technique is easy for opening the medial compartment. Make sure the position of the operating table is high enough.	When using the Knee Arthroscopy Belt for knee arthroscopy with the leg on the table technique, opening the lateral compartment can be more challenging. Shift the leg support of the Knee Arthroscopy Belt a bit backward while rotating your pelvis toward the patient’s knee. Alternatively, use the standard figure-of-4 technique and use the Knee Arthroscopy Belt solely for opening the medial compartment.

It is important to understand that the Knee Arthroscopy Belt is a nonsterile device that is worn under the sterile surgical gown. At all times, the surgical gown should be handled with care when performing surgery with the Knee Arthroscopy Belt. The use and positioning of the Knee Arthroscopy Belt are illustrated in Video 1.

## Discussion

The Knee Arthroscopy Belt is designed to improve ergonomics during arthroscopic procedures of the knee. It provides support to the lumbar spine of the surgeon and helps stabilize the lower leg of the patient during surgery. These characteristics make performing knee arthroscopy easier and with less effort, thus minimizing fatigue.

Possible ergonomic advantages are improving surgeon posture, reducing fatigue, and potentially minimizing musculoskeletal injury risk.

Possible surgical advantages are reduced surgical time, as well as better and more stable fixation of the patient’s leg during procedures. This results in less need for aid of a surgical assistant and more surgical precision. Also, we have experienced improved visualization of the medial and lateral compartments when using the Knee Arthroscopy Bel. The Knee Arthroscopy Belt can also be worn over a lead apron (under the sterile gown).

Possible limitations could include abdominal discomfort when wearing the Knee Arthroscopy Belt. Furthermore, the leg supports can sometimes feel uncomfortable on the ilium of the surgeon when performing surgery with the brackets in certain positions, particularly during varus or valgus stress. When this is the case, the leg supports can be moved a bit to the front or the back, or the surgeon’s pelvis can be rotated somewhat more to the front or the back. The main advantages and disadvantages of the technique are presented in Table 2.

**Table 2:** Advantages and Disadvantages

Advantages	Disadvantages
Improves lumbar spine support during surgery	May cause slight abdominal discomfort
Stabilizes the patient’s lower leg, reducing the need for an assistant	Requires a short learning curve to master
Reduces fatigue and improves posture for the surgeon	Bracket pressure may cause discomfort on the iliac crest with prolonged use
Easy to use in combination with a lead apron (pediatric anterior cruciate ligament or other cases) (Fig 7)	Cannot be used with open wounds around the surgeon’s pelvis/abdomen, during pregnancy (second/third trimester), or under certain medical conditions
Ideal for solo arthroscopy without an assistant	The table height can be higher than you are used to compared to the classic technique with a step stool
The Knee Arthroscopy Belt can be used for knee arthroscopy with a free lower leg, as well as the leg on the table technique	Using the Knee Arthroscopy Belt for knee arthroscopy with the leg on the table technique is easy for opening the medial compartment; opening the lateral compartment can be somewhat of a hassle
We have experienced improved visualization of the medial and lateral compartments when using the Knee Arthroscopy Belt	The Knee Arthroscopy Belt facilitates controlled pressure on the knee when accessing the joint compartments. Nevertheless, care must be taken to avoid applying excessive force, as this may lead to unintended ligamentous injury

Lastly, a short learning curve might be needed to use the Knee Arthroscopy Belt optimally. It could take up to 20 arthroscopic procedures to truly understand the principle. When a surgeon has been using a different technique for years, it takes time to adjust.

## Declaration of Generative AI and AI-Assisted Technologies in the Writing Process

During the preparation of this work, the authors used ChatGPT in order to improve language and readability. After using this tool/service, the authors reviewed and edited the content as needed and take full responsibility for the content of the publication.

## Disclosures

The authors declare the following financial interests/personal relationships which may be considered as potential competing interests: J.S.L. is the owner of Knee Arthroscopy Belt B.V., is the patent holder of the Knee Arthroscopy Belt, and has equity or stocks with Knee Arthroscopy Belt B.V. W.A.v.d.W. is the owner of Knee Arthroscopy Belt B.V. and has equity or stocks with Knee Arthroscopy Belt B.V. The other author (D.E.M.) declares that they have no known competing financial interests or personal relationships that could have appeared to influence the work reported in this paper.

## Supplementary Data

Video 1 Demonstration of the correct use and positioning of the Knee Arthroscopy Belt during knee Arthroscopy.
